# Revised Standardized Equation for Hydrogen Gas Densities for Fuel Consumption Applications

**DOI:** 10.6028/jres.113.028

**Published:** 2008-12-01

**Authors:** Eric W. Lemmon, Marcia L. Huber, Jacob W. Leachman

**Affiliations:** National Institute of Standards and Technology, Boulder, CO 80305-3337; University of Wisconsin-Madison Cryogenics Lab

**Keywords:** density, equation of state, fuel consumption, hydrogen, pressure, temperature

## Abstract

An equation for the density of hydrogen gas has been developed that agrees with the current standard to within 0.01 % from 220 K to 1000 K with pressures up to 70 MPa, to within 0.01 % from 255 K to 1000 K with pressures to 120 MPa, and to within 0.1 % from 200 K to 1000 K up to 200 MPa. The equation is a truncated virial-type equation based on pressure and temperature dependent terms. The density uncertainty for this equation is the same as the current standard and is estimated to be 0.04 % (combined uncertainty with a coverage factor of 2) between 250 K and 450 K for all pressures, and 0.1 % for lower temperatures. Comparisons are presented with experimental data and with the full equation of state.

## 1. Introduction

The equilibrium temperature and pressure of a gas before and after usage within a storage tank of known, and essentially fixed, volume can be used to calculate consumption. Equations of state for calculating the thermodynamic properties generally provide the pressure as a function of density and temperature. In some fuel consumption applications, this form is inconvenient to use since the equation must be solved in an iterative manner in order to provide the density in terms of pressure and temperature. In order to easily calculate gaseous hydrogen fuel consumption in vehicle applications in which temperature and pressure are measured, an equation with these independent properties that agrees with the current standard is desirable.

The former standard for hydrogen was the equation of state by Younglove [1] published in 1982 and was a 32-term expression for pressure as a function of temperature and density, *p* (*T*, *ρ*). This equation for parahydrogen, adapted for use for normal hydrogen, was once considered adequate for density calculations in the region of interest. It is a rather dated standard, and the basic source of the parahydrogen equation is a 1975 National Bureau of Standards (NBS) technical report [2,3]. (NBS is the former name of the National Institute of Standards and Technology, NIST.) The density uncertainty for this equation was estimated as 0.2 % (combined uncertainty with a coverage factor of 2). Some of the older NBS material and a bibliography of hydrogen property information can be found through the NIST hydrogen web site http://www.nist.gov/public_affairs/hydrogen.htm that is part of the larger hydrogen site for the United States Government, http://www.hydrogen.gov.

There have been several recent advances in the state of the art for the thermodynamic properties of hydrogen. New equations of state have been developed in the Master’s Thesis of Jacob Leachman [4] for normal hydrogen, parahydrogen, and orthohydrogen; these equations will be fully documented elsewhere [5]. These equations replace those presented by Younglove in 1982 and decrease the uncertainty in density in the normal hydrogen equation of state from 0.2 % to 0.04 % in the range from 250 K to 450 K with pressures up to 300 MPa.

In 2006, Lemmon et al. [6] published a short equation for the density of hydrogen between 220 K and 400 K with pressures to 45 MPa. This equation calculated density as a function of temperature and pressure and agreed with the Younglove equation of state to less than 0.01 % in density. It is used in SAE Standard J2572, Recommended Practice for Measuring Fuel Consumption and Range of Fuel Cell and Hybrid Fuel Cell Vehicles Fuelled by Compressed Gaseous Hydrogen [6]. With the new equation of state of Leachman, the 2006 work is now out of date and a replacement is needed. The work presented here updates and expands the 2006 work and provides an equation for the density of hydrogen as a function of temperature and pressure, expanding the temperature range to the upper limit used by Leachman and the pressure range to 200 MPa. The 2006 work gave additional information on the use of the equation in hydrogen gas consumption applications that is not repeated here.

## 2. Hydrogen Equation as a Function of Pressure and Temperature

The Leachman equation of state from Ref. [4] is a 14-term Helmholtz energy equation written as

$$\begin{array}{l} \alpha^{r}(\delta ,\tau )=\sum N_{k}\delta^{d_{k}}\tau^{t_{k}}+\sum N_{k}\delta^{d_{k}}\tau^{t_{k}}\exp(-\delta^{l_{k}})\\ \;+\sum N_{k}\delta^{d_{k}}\tau^{t_{k}}\exp\left(\eta {\left(\delta -\varepsilon_{k}\right)}^{2}+\beta {\left(\tau -\gamma_{k}\right)}^{2}\right) \end{array}$$ (1)

where *α*^r^ is the residual Helmholtz energy, *δ* is the reduced density *ρ*/*ρ*_c_, *τ* is the reduced inverse temperature *T*_c_/*T*, and the subscript c denotes the value at the critical point. The other parameters are constants that were determined during the fitting of available property data. Values of the constants can be found in Refs. [4,5]. This expression, together with an equation for the ideal gas heat capacity, enables a thermodynamically consistent calculation of many properties of hydrogen in the liquid, vapor, and supercritical phases (density, isochoric and isobaric heat capacities, sound speed, phase boundaries, enthalpy, etc.) from about 14 K to 1000 K with pressures to 2000 MPa. This equation is implemented in the REFPROP software [7] and is available through the NIST Chemistry Webbook (http://webbook.nist.gov). Inversion of the equation through iterative solutions is straightforward; nonetheless, direct use of Eq. (1) for hydrogen consumption calculations may not be convenient.

A common equation for the density of gases is based on the virial series, which has a statistical-mechanical basis in terms of the relation between the number of particles interacting and the significance of such multiparticle interactions at a particular gas density. Often, the virial equation is written in the form of an expression for the pressure as a sum of the powers of density multiplied by temperature-dependent virial coefficients. Alternatively, the temperature-dependent virial equation may be expressed in terms of the powers of pressure. For the compressibility factor, *Z* = *p* / (*ρRT*), this becomes

$$Z(p,T)=1+\sum_{i=2}^{n}B_{i}^{*}(T)p^{i-1},$$ (2)

where the B^*^_i_(*T*) quantities are the temperature-dependent pressure virial coefficients.

The lower virial coefficients (e.g., the second virial coefficient, B^*^_2_) can be calculated theoretically if the interaction potential between hydrogen molecules is known (e.g., through quantum mechanical calculations). However, the current effort has focused on establishing an equation of the form given in Eq. (2) that agrees with the standard of Ref. [4] to within 0.01 % in density over the range of interest. No attempts were made to determine the virial coefficients theoretically, although experimental values of the second virial coefficient were used in the development of the formulation in Ref. [4].

The specific terms and coefficients were determined by calculating a set of compressibility factor values distributed in (*p*, *T*) space with the REFPROP [7] implementation of Eq. (1). These values were then used in a structural optimization/regression algorithm [8] with the system constrained to consider the lowest virial coefficients of Eq. (2) and simple temperature dependences for B^*^_i_ (*T*). This structure/parameter space was searched systematically until an equation meeting the criterion of 0.01 % agreement in density was obtained. Virial coefficients up to the sixth power were considered in order to match the isothermal curvature of the hydrogen equation of state above 150 K. Initial fitting considered only integer values of the exponents on pressure. However, it was only possible to achieve high accuracy and expand the range of coverage by using noninteger values. The use of noninteger exponents has a negative impact on only the third and higher derivatives of the equation, which is not of concern in this work. The resulting coefficients and exponents were truncated to the extent allowed to retain agreement with the equation of state within the specified tolerance. The equation was examined to ensure reasonable extrapolation over a broader temperature and pressure range; however, derivatives were not examined as was done with the original equation of state. The equation given here is intended for use only in density calculations over the range specified. The underlying equation of state [4] should be used for all other calculations.

The resulting expression for the compressibility factor is

$$Z(p,T)=\frac{p}{\rho RT}=1+\sum_{i=1}^{9}a_{i}{\left(\frac{100K}{T}\right)}^{b_{i}}{\left(\frac{p}{1\text{MPa}}\right)}^{c_{i}}.$$ (3)

The constants associated with Eq. (3) are given in Table 1. The equation and its constants are given for pressure in megapascals (MPa) and temperature in kelvins (K). The mass of diatomic hydrogen and the molar gas constant given in Table 1 are from the most recent tabulations of such information [9,10]. The molar mass is given to aid in conversions from molar to mass density. Test points that can be used to verify algorithms based on Eq. (3) are given in Table 2. More digits are given than the uncertainty in the equation to aid in validation.

## 3. Evaluation of the Equation

As discussed above, Eq. (3) and the related constants were established by calculating values from the equation of state [4] and regressing the structure and coefficients using these values. Equation (3) was in turn evaluated through a more thorough comparison with the equation of state and by comparing with available experimental data for the density of hydrogen over the range of the equation.

Densities were computed with the new formulation (Eq. (3) with constants from Table 1) and compared with densities obtained from the equation of state of Leachman [4] for several temperature and pressure ranges to assess how well Eq. (3) agrees with the equation of state. Of specific interest is the (*T, p*) region where densities calculated from Eq. (3) and densities from the full equation of state of Leachman are in agreement to within 0.01 %. One million points in the (*T, p*) region from 220 K to 1000 K with pressures to 70 MPa, and a second (*T, p*) region from 255 K to 1000 K with pressures to 120 MPa, were randomly generated and the results from Eq. (3) were compared with the densities computed from the Leachman equation of state with the REFPROP software [7]. For both of these (*T, p*) regions the randomly selected points never exceeded 0.01 % deviation in density from the equation of state of Leachman [4]. Over the range of temperatures from 220 K to 1000 K with pressures to 70 MPa, the average absolute percentage deviation for these million points was 0.0020 %, with a standard deviation of 0.0022 %. The maximum positive deviation was 0.0075 %, and the largest negative deviation was – 0.0046 %. The largest deviations occurred at the low temperature limit of 220 K. If the temperature range is limited to 255 K to 1000 K, Eq. (3) is in agreement to within 0.01 % with the equation of state up to 120 MPa. Over this (*T, p*) range, the average percentage deviation for these million points was 0.0025 %, with a standard deviation of 0.0024 %. The maximum positive deviation was 0.0097 %, and the largest negative deviation was −0.0026 %.

**Fig. 1:**
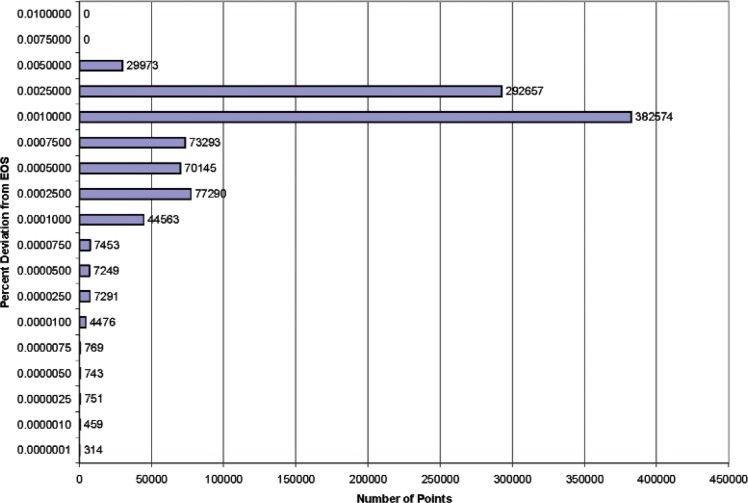
Histogram illustrating the frequency of percentage density deviations for a sample containing one million points over the range of temperatures from 220 K to 1000 K with pressures to 70 MPa.

**Fig. 2:**
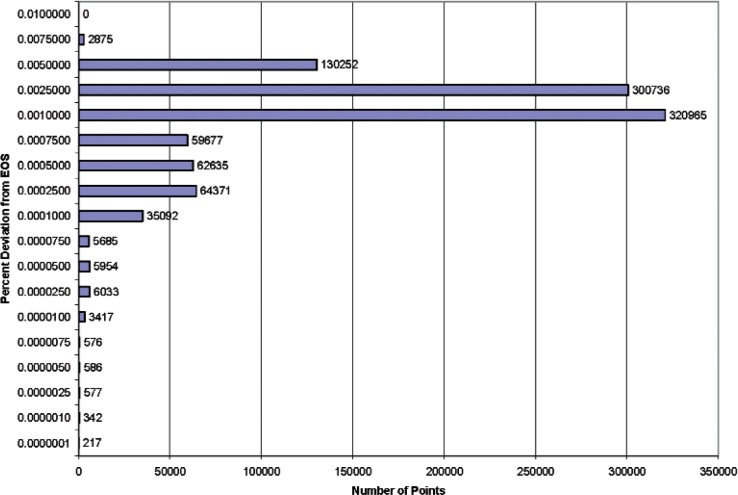
Histogram illustrating the frequency of percentage density deviations for a sample containing one million points over the range of temperatures from 255 K to 1000 K with pressures to 120 MPa.

Figures 1 and 2 are histograms of the results. Figure 1 covers the region 220 K to 1000 K at pressures up to 70 MPa, while Fig. 2 displays the range 255 K to 1000 K at pressures to 120 MPa. Figure 1 shows that the largest number of points had a percentage deviation between 0.001 % and 0.0025 %; the second largest group had deviations between 0.0025 % and 0.005 %. There were no points with deviations larger than 0.0075 %. Figure 2 shows that the largest number of points had a percentage deviation between 0.001 % and 0.0025 %; the second largest group had deviations between 0.0025 % and 0.005 %. There were no points with deviations larger than 0.01 %.

**Fig. 3:**
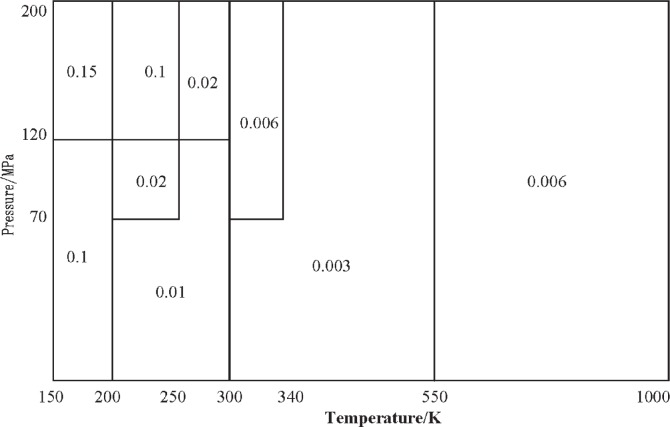
Maximum percent differences between the equation of state of Leachman [4] and the equation presented here (axes are not to scale).

The new formulation (Eq. (3) with constants from Table 1) can be extrapolated outside the two ranges discussed above. In the extended region with pressures to 200 MPa but with temperatures higher than 300 K, the maximum deviation (as compared to the equation of state of Leachman [4]) is less than 0.006 %. If the lower temperature limit is lowered to 200 K but with pressures still up to 200 MPa, the maximum deviation is 0.1 %, and if the lower temperature limit is lowered to 150 K, the maximum deviation is 0.15 %. Figure 3 shows this evaluation in more detail.

**Fig 4:**
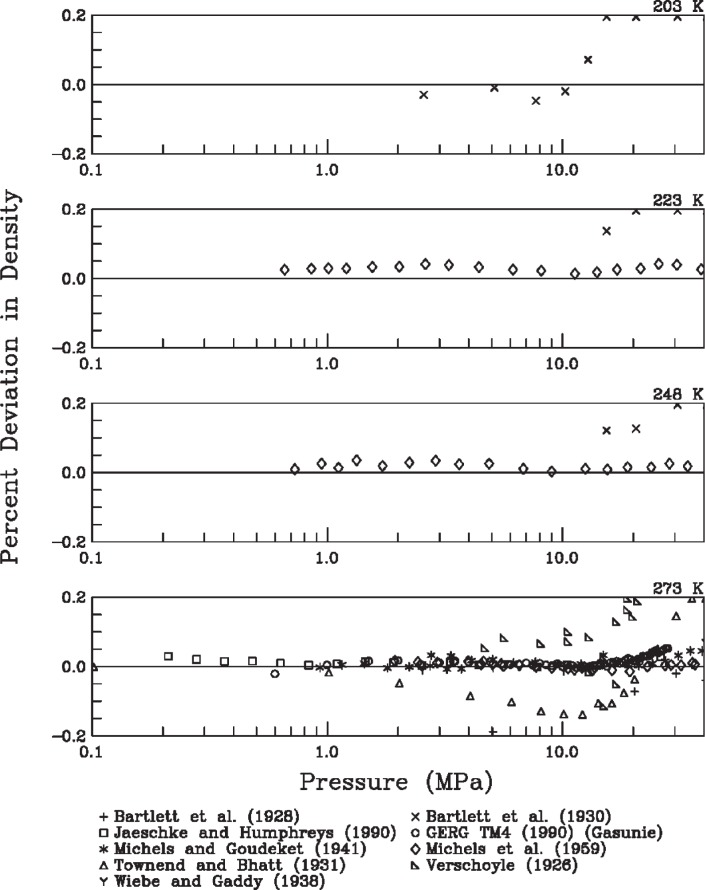
Density deviations as a function of pressure for temperatures from 203 K to 273 K.

**Fig. 5:**
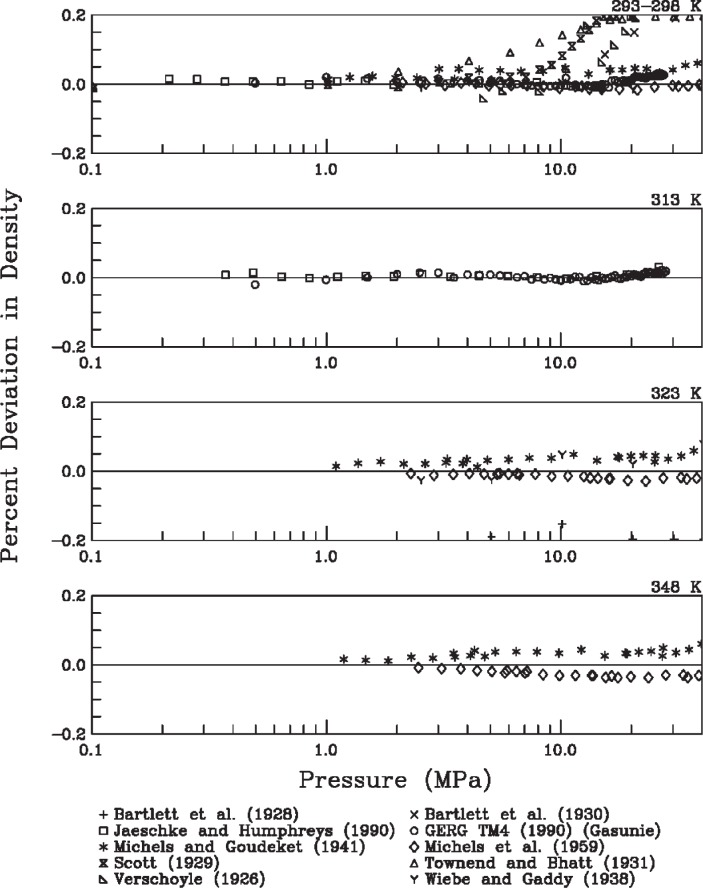
Density deviations as a function of pressure for temperatures from 293 K to 348 K.

**Fig. 6:**
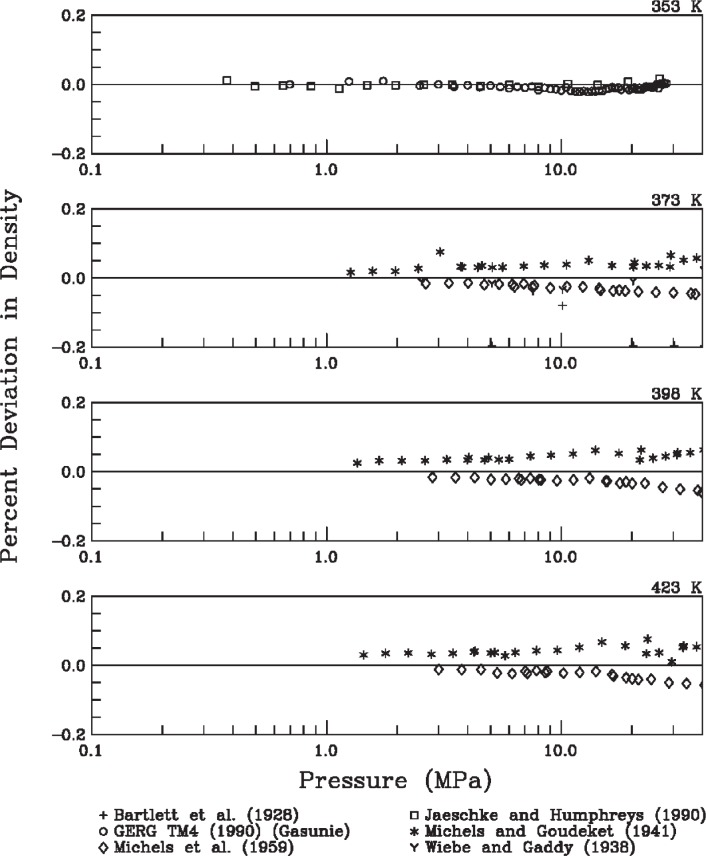
Density deviations as a function of pressure for temperatures from 353 K to 423 K.

The second type of evaluation was direct comparison with available experimental data. Figures 4 to 6 show the deviations between densities calculated with Eq. (3) and experimental data in the literature [11–19]. The subplots in these figures include data from various sources grouped in isotherms. A line comparing the representation of Eq. (3) with the equation of state of Leachman [4] is not provided, as it is extremely close to the zero line (within 0.01 %, as indicated above).

Several observations can be made from the deviation plots. These figures generally support the uncertainty estimate for the equation of state of Leachman [4]. The estimate of 0.04 % as a combined uncertainty with coverage factor of two seems reasonable in this range considering only the scatter of the available density data and the agreement between the equation and these data. These observations support the use of Eq. (3) for fuel consumption calculations based on density calculations from initial and final pressures and temperatures.

## 4. Conclusions

Fuel consumption can be determined in multiple ways. From a regulatory view, it is mandatory to have consistent methods that can be used for independent verification. Equivalent measurement methods allow maximum flexibility in the regulated industry and confidence in data integrity. The determination of the mass of hydrogen gaseous fuel use is quite straightforward after the initial and final temperatures, pressures, and volumes have been accurately established. Consistent use of an expression for mass determination requires a standard method by which the compressibility factor can be calculated.

Equation (3) can form the basis of such a standard. It was developed to provide consistency with the calculations from a NIST Standard Reference Database [7], and it has been shown to provide reasonable agreement with the currently available data. The simple form of Eq. (3) agrees with the standard of Ref. [4] to within 0.01 % from 220 K to 1000 K with pressures up to 70 MPa, and from 255 K to 1000 K with pressures up to 120 MPa, although the estimated uncertainty of the resulting hydrogen density is 0.04 %. The equation can be extended from 150 K to 1000 K with pressures up to 200 MPa with an uncertainty of 0.15 % at the lowest temperatures. The equation is not adequate for the calculation of other hydrogen properties. There might be other uncertainties in the quantification of hydrogen fuel consumption that have not been explicitly considered here. These could include the temperature and pressure dependence of the tank volume, equilibration of the temperature and pressure measurements, and other uncertainties in the measurement of temperature and pressure.

## Figures and Tables

**Table 1:** Constants associated with the density equation for normal hydrogen

*i*	*a_i_*	*b_i_*	*c_i_*
1	0.058 884 60	1.325	1.0
2	−0.061 361 11	1.87	1.0
3	−0.002 650 473	2.5	2.0
4	0.002 731 125	2.8	2.0
5	0.001 802 374	2.938	2.42
6	−0.001 150 707	3.14	2.63
7	0.958 852 8 × 10^−4^	3.37	3.0
8	−0.110 904 0 × 10^−6^	3.75	4.0
9	0.126 440 3 × 10^−9^	4.0	5.0

Molar Mass: *M* = 2.015 88 g/mol

Universal Gas Constant: *R* = 8.314 472 J/(mol · K)

**Table 2:** Test points for validating computer code based on Eq. (3)

*T*(K)	*p*(MPa)	*Z*	*ρ* (mol/1)
200	1	1.00675450	0.59732645
300	10	1.05985282	3.78267048
400	50	1.24304763	12.09449023
500	200	1.74461629	27.57562673
200	200	2.85953449	42.06006952
